# Pulse Duration as Well as Current Direction Determines the Specificity of Transcranial Magnetic Stimulation of Motor Cortex during Contraction

**DOI:** 10.1016/j.brs.2016.09.008

**Published:** 2017

**Authors:** Ricci Hannah, John C. Rothwell

**Affiliations:** Sobell Department of Motor Neuroscience and Movement Disorders, UCL Institute of Neurology, London, UK

**Keywords:** Pulse duration, Current direction, Short latency afferent inhibition, Cerebellum, Transcranial direct current stimulation, AP, anterior–posterior, cTMS, controllable pulse parameter transcranial magnetic stimulation device, CSN, corticospinal neuron, MEP, motor evoked potential, PA, posterior–anterior, tDCS_Cb_, transcranial cerebellar direct current stimulation

## Abstract

**Background:**

Previous research suggested that anterior–posterior (AP) directed currents induced by TMS in motor cortex (M1) activate interneuron circuits different from those activated by posterior–anterior currents (PA). The present experiments provide evidence that pulse duration also determines the activation of specific interneuron circuits.

**Objective:**

To use single motor unit (SMU) recordings to confirm the difference in onset latencies of motor-evoked potentials (MEPs) evoked by different current directions and pulse durations: AP_30_, AP_120_, PA_30_ and PA_120_. To test whether the amplitude of the MEPs is differentially influenced by somatosensory inputs from the hand (short-latency afferent inhibition, SAI), and examine the sensitivity of SAI to changes in cerebellar excitability produced by direct current stimulation (tDCS_Cb_).

**Methods:**

Surface electromyograms and SMUs were recorded from the first dorsal interosseous muscle. SAI was tested with an electrical stimulus to median or digital nerves ~20–25 ms prior to TMS delivered over the M1 hand area via a controllable pulse parameter TMS (cTMS) device. SAI was also tested during the application of anodal or sham tDCS_Cb_. Because TMS pulse specificity is greatest at low stimulus intensities, most experiments were conducted with weak voluntary contraction to reduce stimulus threshold.

**Results:**

AP_30_ currents recruited the longest latency SMU and surface MEP responses. During contraction SAI was greater for AP_30_ responses versus all other pulses. Online anodal tDCS_Cb_ reduced SAI for the AP_30_ currents only.

**Conclusions:**

AP_30_ currents activate an interneuron circuit with functional properties different from those activated by other pulse types. Pulse duration and current direction determine what is activated in M1 with TMS.

## Highlights

• Selective stimulation of inputs to corticospinal neurons may be achieved by manipulating current direction and pulse duration.
• Neural populations recruited by brief (30 μs) anterior–posterior currents exhibited the greatest sensitivity to somatosensory input.
• Pulse duration is an important determinant of what is activated with TMS in human motor cortex.

## Introduction

**Figure 1:**
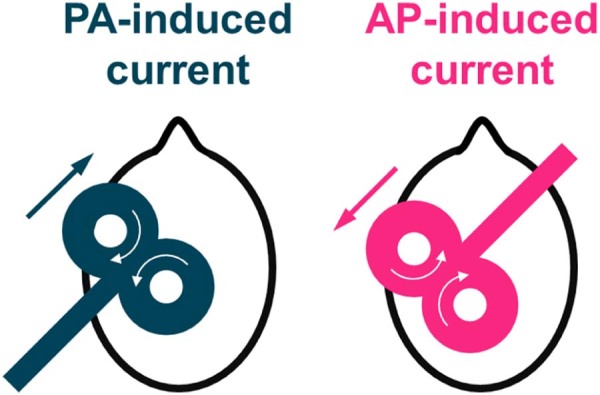
A schematic representation of the TMS coil orientations used. Straight arrows indicate the direction of the current induced in the brain, whilst curved arrows indicate the direction of current in the TMS coil. Posterior–anterior (PA) induced currents in the brain were produced by the coil being oriented posterolaterally at an angle of ~45° to the midline, and anterior–posterior (AP) induced currents in the brain were elicited by placing the coil 180° to the PA currents [2], [5], [6], [7], [8].

A single TMS pulse over primary motor cortex (M1) activates the axons of excitatory synaptic inputs to corticospinal neurons (CSNs), which initiates descending activity in the corticospinal tract and eventually produces a motor evoked potential (MEP) in contralateral muscles [1]. It is well known that the orientation of the current induced across the central sulcus influences the activation of the CSN [2], [3], [4]. Day et al. originally showed that posterior–anterior (PA; Fig. 1) induced-currents gradually recruited indirect wave (I-wave) inputs in order of their appearance (I_1_, I_2_, I_3_ etc.), whilst anterior–posterior (AP) currents preferentially recruited late inputs (I_3_) [2], implying that the early (I_1_) and late (I_3_) I-waves might therefore reflect activity of different excitatory inputs. However, recent accounts suggest that the situation may be slightly more complicated. Ni et al. evaluated the effects of somatosensory inputs from the hand on MEPs (short-latency afferent inhibition, SAI) evoked by different current directions, and found SAI suppressed I_3_ waves recruited by PA currents more readily than I_3_ waves recruited by AP currents [8]. They concluded that the late I-waves activated by PA and AP current directions were generated by different excitatory inputs. This finding was consistent with recordings of corticospinal activity evoked by AP and PA currents, showing that although both orientations produce I_1_, I_2_ and I_3_ waves, the peaks are slightly delayed and more dispersed for AP pulses compared to PA pulses.

**Figure 2:**
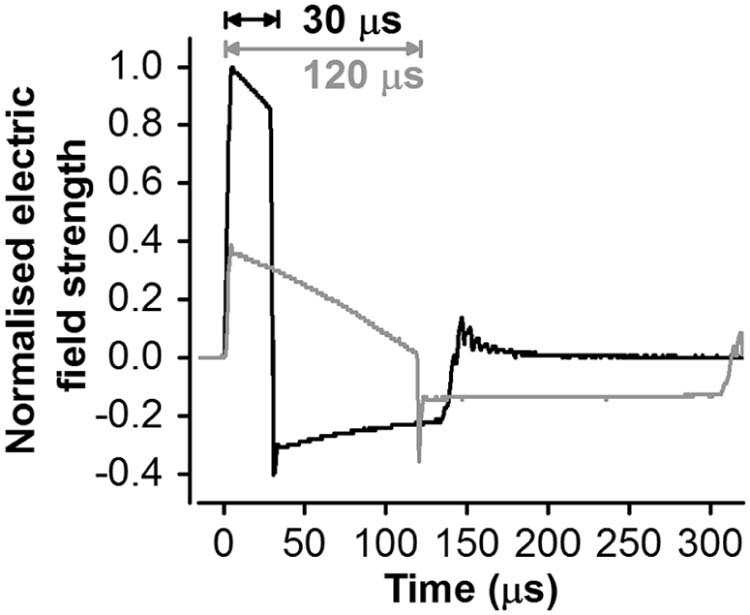
cTMS electric field pulse waveforms for pulse durations of 30 and 120 µs, referring to the duration of the first dominant phase of the electric field, recorded with a search coil and normalised to the maximum amplitude recorded with the 30 µs pulse. The pulse amplitude was limited by the cTMS device to 100 and 37 percent of maximum amplitude for 30 and 120 µs pulses, respectively [9], [10].

Using a novel controllable pulse parameter TMS (cTMS; [9]) device, which permits control of the stimulus pulse duration (Fig. 2), we recently found short duration (30 µs) AP currents (i.e. AP_30_) produced longer latency MEPs than more standard long duration (120 µs) AP (AP_120_) currents, and thus appeared to activate axons with a delayed input to CSNs [10] (for comparison, traditional pulses are ~82 µs in duration [11]). We had assumed that AP_30_ and AP_120_ currents stimulated the axons of same long latency inputs, but that AP_30_ did so more selectively without also recruiting earlier inputs. Here we tested the hypothesis that the inputs recruited by PA, AP_120_ and AP_30_ currents might actually represent independent circuits by assessing whether they had different functional properties. To do this we evaluated the effects of SAI on MEPs evoked by different combinations of pulse duration and current direction.

Different lines of evidence suggest that the interaction of afferent input with M1 is affected by cerebellar function. First, patients with cerebellar degeneration [12] and Alzheimer's disease [13] exhibit abnormal SAI, and in the latter this is partially restored after cerebellar theta burst stimulation. Second, modulation of cerebellar activity using transcranial direct current stimulation over the cerebellum (tDCS_Cb_) has been reported to reduce the size of AP-evoked, but not PA-evoked, MEPs when assessed during voluntary muscle activation [7]. We therefore tested whether cerebellar excitability changes specifically interacted with SAI evaluated with AP_120_, AP_30_ or PA_120_ test pulses.

## Methods

### Subjects

Twenty-seven volunteers (15 males; age 28 ± 6 years; 25 right-handed), who reported no contraindications to TMS [14], provided written informed consent prior to participating in the study which was approved by University College London Ethics Committee.

### Surface electromyogram (EMG)

Surface EMG electrodes (WhiteSensor 40713, Ambu®, Denmark) were placed in a belly-tendon arrangement over the first dorsal interosseous (FDI) and abductor pollicis brevis (APB) muscles of the dominant hand. The ground electrode was over the wrist. Signals were amplified with a gain of 1000 (Digitimer, UK), band-pass filtered (5–3000 Hz), digitised at 5 kHz (1401; CED, Cambridge, UK), and analysed with Signal v5.10 software.

### Single motor unit (SMU) EMG

SMU EMG activity was recorded from the FDI of the dominant hand via concentric needle electrodes (25 × 0.3 mm; Ambu®, Denmark). Signals were amplified with a gain of 10,000, band-pass filtered (60 Hz–10 kHz), and sampled at 10 kHz using the same hardware and software as for surface EMG recordings. Auditory and visual feedback of EMG activity helped the subject to maintain the motor unit firing at ~10 Hz.

### Transcranial magnetic stimulation (TMS)

MEPs in the dominant FDI were evoked using a custom built cTMS device (cTMS3; Rogue Research Inc., Canada) [9], connected to a standard figure-of-eight coil (wing diameter 70 mm; Magstim, UK). Four combinations of TMS current direction and pulse duration (PA and AP; 30 and 120 µs; Figure 1, Figure 2) were applied: AP_30_, AP_120_, PA_30_, and PA_120_. The motor hot spot was found by searching for the position where slightly suprathreshold PA_120_ currents produced the largest and most consistent MEP in FDI. The position was marked on a cap worn by the participants.

In experiment 1, the test stimulus (TS) intensity required to produce a small increase (~10%) in the SMU firing probability was determined for each TMS pulse type. Otherwise, TS intensity was defined as that required to produce a 1 mV MEP determined either during background contraction (~10% maximum EMG amplitude) (experiments 2 and 4) or at rest (experiment 3). Pulses were given every 3 s (experiment 1) or every 4–5 s (experiments 2–4).

### Electrical stimulation

Conditioning stimuli (CS), square wave (0.2 ms) pulses, were delivered to the median nerve at the wrist or to digital nerves of the index and middle fingers via bipolar cup or ring electrodes (cathode proximal) [15], respectively, connected to a constant-current stimulator (DS7AH, Digitimer, UK). Median nerve intensity was just above motor threshold (0.2 mV APB M-wave; Table 2); digital nerve intensity was three times the sensory threshold (Table 2).

### Short latency afferent inhibition

The inter-stimulus intervals (ISI) between electrical CS and TMS were set according to individual N20 somatosensory-evoked potential latency [15], for median or digital nerve stimulation (Table 2). Electrical stimuli preceded TMS by N20 +2 and +4 ms. The four different TMS pulses were tested in separate blocks, consisting of TMS TS delivered alone or randomly preceded by CS. The order of TMS pulse types was randomised for each participant, and each block was separated from the next by 3 min relaxation.

### Cerebellar transcranial direct current stimulation (tDCS_Cb_)

Direct current stimulation (2 mA) was applied (Magstim, UK) as described previously [7], [16], [17]. The anode was 3 cm lateral to the inion, ipsilateral to the dominant hand, with the cathode over the buccinator muscle on the same side. Real anodal tDCS_Cb_ (tDCS_Cb-Anodal_) was delivered for 27 minutes. Sham tDCS_Cb_ (tDCS_Cb-Sham_) involved ramping the current up and down and down for 30 s each at the start and end of the stimulation period, but remained off for the remainder of the 27 minutes.

### Experimental design

#### Experiment 1: single motor unit response latencies to different TMS pulses

Complete data sets were obtained from 7 SMUs from 7 participants. TMS TS were triggered within 65–85 ms of the previous SMU spike in order to maximise the likelihood of evoking a response (see Ref. [18]). Sweeps were triggered every 3 seconds (±10%), and at least 100 sweeps were recorded per TMS pulse type, with a 5 minute rest being given between conditions.

#### Experiment 2: sensitivity of SAI evoked in active muscle to different TMS current directions and pulse durations

Complete datasets were obtained in 21 participants. Experiments were performed during weak contraction so that MEPs could be evoked by low intensity stimulation. The differences between pulse types in the latency of evoked responses are obscured at higher intensity, as needed at rest (see experiment 3), since pulses then recruit a mixture of inputs [1], [2], [3]. Sixty trials were obtained for each TMS pulse type (20 TS alone; 20 at each of the two CS–TS ISIs). Trials were delivered in 3 sets of 20, with a 1 minute rest between sets to avoid fatigue.

In a subset of 10 individuals we compared SAI evoked by median nerve (mixed) and digital nerve stimulation (cutaneous), to test whether any effects of TMS pulse type on SAI were dependent on the type of afferent input. Twenty responses were recorded for TS alone and twelve each of the median and digital nerve conditioned responses at each ISI (total 68 trials per TMS pulse type).

#### Experiment 3: measuring SAI (median nerve) at rest

SAI was tested at rest to evaluate whether higher stimulus intensities obscure the effect of TMS pulse type on SAI seen with more selective, low intensity pulses. We acknowledge that compared to experiment 2 we have changed both the stimulus intensity (active 1 mV vs. resting 1 mV) and state (active vs. resting muscle) at the same time, and thus we are not directly evaluating the influence of stimulus intensity on SAI. However, the average intensity used to evoke the 1 mV MEP in active muscle (Table 1) was approximately equivalent to resting motor threshold (and 18–28% lower in relative terms than resting 1 mV intensity). The MEPs evoked by this intensity at rest would have been too small to analyse. It would be worth exploring in future whether the results we obtained can be replicated with low intensity (e.g. 0.5 mV) pulses at rest. Additionally, resting MEP threshold for AP stimulation requires high amplitude pulses [10]. Thus, because of limitations in cTMS output (Fig. 1), we were only able to examine SAI at rest in 8 individuals from experiment 2.

#### Experiment 4: effects of tDCS_Cb_ on SAI (median nerve) during contraction

Experiment 2 was repeated on 11 participants (7 had participated in Exp. 2) during concurrent real or sham tDCS_Cb_. The tDCS_Cb-Anodal_ and tDCS_Cb-Sham_ sessions were conducted randomly >3 days apart. SAI was assessed twice during each session, prior to (Off) and during (On) tDCS_Cb_. The rationale for testing online, rather than after tDCS_Cb_, was that a previous study reported online effects of tDCS_Cb-Anodal_ on AP-evoked MEPs [7].

Given that the MEP latency and SAI for PA-currents was unaffected by pulse duration (Exp, 2), this experiment used AP_30_, AP_120_ and PA_120_. For each of the three TMS pulse types, responses to fifteen TS and fifteen of each CS–TS interval (i.e. 45 trials in total) were recorded in each tDCS_Cb_ state (Off and On), with the order in which TMS pulse types were assessed being counter-balanced. Within each TMS pulse type, trials were delivered in 3 sets of 15 (5 TS alone, 10 conditioned), with 1 minute rest between sets to avoid fatigue. Measurements started 5 minutes after tDCS_Cb_ application in order to allow any effects to build up.

### Data analyses

**Figure 3:**
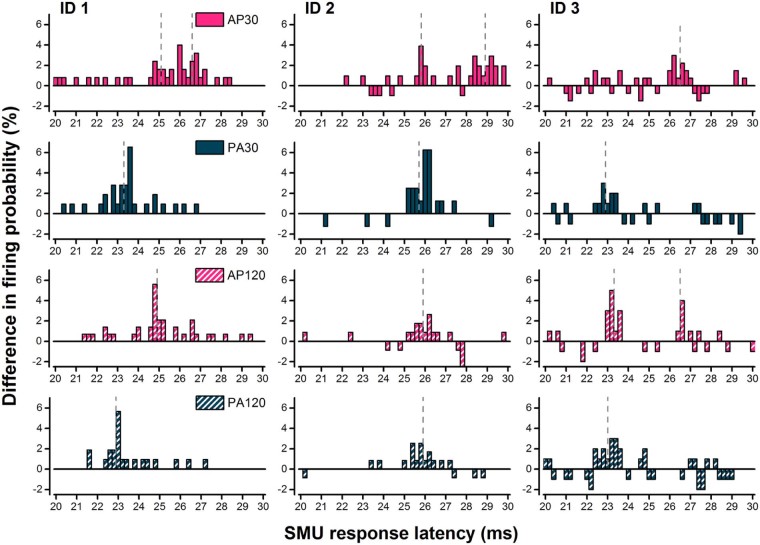
Post-stimulus time histograms (PSTH) for three individuals (each shown in a different column: ID 1, ID 2, ID 3) constructed from the difference between control PSTH (pre-stimulus; not shown) and TMS-evoked PSTH and normalised to the number of trigger pulses. The x-axis indicates the time after the TMS stimulus and the y-axis indicates the difference in firing probability between the two PSTHs. 1st row relates to AP_30_, 2nd row to PA_30_, 3rd to AP_120_ and 4th to PA_120_ currents. Note dashed grey lines indicate the latency of identified peaks. AP_30_ currents generally evoked a peak ~3 ms later than the earliest peak evoked by PA currents, though it was sometimes accompanied by an earlier peak (see ID 1 and ID 2).

For Exp. 1, the control post-stimulus time histogram (PSTH) of the distribution of firing times driven by volition was subtracted from the TMS-evoked PSTH reflecting the change in distribution of firing caused by TMS. The resulting PSTH (0.2 ms bin size) was normalised to the number of trigger pulses to give the change in firing probability (Fig. 3) [18]. The latency of each peak of increased firing probability was measured as follows. A peak was defined as an increase in firing probability of four or more percent in two or more adjacent time bins [2]. The latency of a peak was defined as the average time of the onset and offset, the identification of which were aided via use of the cumulative sum procedure [19]. Where more than one peak was observed, the latency of the two peaks was averaged to produce a weighted mean based on the relative size (increase in firing probability) of the peaks.

MEP onset latencies in response to TS alone from Exp. 2 were measured via visual inspection on a trial-by-trial basis [10], [20] for each TMS pulse type, and subsequently averaged across trials. Differences in the mean latency of SMU peaks and surface MEPs were calculated for each TMS pulse type in each individual and averaged.

In Exp. 2–4, peak-to-peak MEP amplitudes were measured on a trial-to-trial basis and used to calculate a mean. SAI was expressed as a ratio of conditioned to test MEP amplitudes, i.e. the amplitude of the conditioned MEP divided by the amplitude of the test MEP. Ratios <1 indicated inhibition and values >1 indicated facilitation.

### Statistical analyses

Repeated measures ANOVA (rmANOVA) was used to evaluate the majority of the data. The two main factors were current direction (AP, PA) and pulse duration (30, 120 µs) for (1) mean SMU peak latencies (experiment 1); (2) MEP onset latencies (experiment 2); and (3) TS intensities (i.e. the threshold for producing a given response size; experiments 1–3).

For experiments 2–3, the same main factors (current direction and pulse duration) were used in an analysis to confirm similar absolute size of non-conditioned TS MEPs. Thereafter, a three-way rmANOVA [current direction (AP, PA); pulse duration (30, 120 µs); ISI (N20+2, N20+4)] was performed on the normalised amplitude of conditioned MEPs to investigate differences in SAI between TMS pulse types. Separate ANOVAs were performed for SAI (median) and SAI (digital). In the absence of any effects of ISI, further two-way rmANOVAs were performed after averaging ISI data. *Post hoc* comparisons were made with Bonferroni-corrected paired *t*-tests (i.e. *P* values obtained from statistical analyses were multiplied by *N* comparisons and compared with the critical *P* value of 0.05).

For experiment 4, tDCS_Cb-Anodal_ and tDCS_Cb-Sham_ effects on SAI were first examined separately. A two-way rmANOVA [TMS pulse type (AP_30_, AP_120_ and PA_120_); tDCS_Cb_ state (Off, On)] confirmed similar TS intensity and absolute size of non-conditioned TS MEPs. Subsequent three-way rmANOVAs were performed on the normalised amplitude of conditioned MEPs [TMS pulse type (AP_30_, AP_120_ and PA_120_); tDCS_Cb_ state (Off, On); ISI (N20+2, N20+4)]. In the absence of any effects of ISI, further two-way rmANOVAs were performed after averaging ISI data. *Post hoc* comparisons were made with Bonferroni corrected *t*-tests to evaluate (1) difference between tDCS_Cb_ states for each TMS pulse type, and (2) differences between TMS pulse types for each tDCS_Cb_ state. Finally, the percentage change in conditioned MEP amplitude from Off to On was calculated for each TMS pulse type, and two-way rmANOVA was performed after averaging across ISI [TMS pulse type (AP_30_, AP_120_ and PA_120_); tDCS_Cb_ state (Off, On)]. *Post hoc* comparisons were made with Bonferroni-corrected paired *t*-tests to compare tDCS_Cb-Anodal_ and tDCS_Cb-Sham_ for each pulse type.

Data are reported as group mean ± SEM. *P* values <0.05 were considered significant. Where necessary, the Greenhouse–Geisser procedure was applied to correct for violations of sphericity in ANOVA. Detailed ANOVA results are shown in Table 3.

## Results

### TS intensities and absolute MEP amplitudes

As shown in Table 1, AP currents required higher stimulus intensities than PA currents (1.2–1.3 times greater for a given pulse width), and narrow TMS pulses required higher stimulus intensities than wider pulses (>2 times greater for a given current direction), particularly for the AP direction. The stimulus intensities used for AP_30_ currents were approximately three times greater compared with those used for PA_120_ currents. These differences are to be expected from the steepness of the strength–duration curves at short pulse durations [10]. This was confirmed using rmANOVAs on the intensity data from experiments 1–3 which showed a significant current direction × pulse duration interaction for all 3 experiments (experiment 1, *F* = 22.1_[1,6]_, *P* = 0.003; experiment 2, *F* = 129.3_[1,20]_, *P *<* *0.001; experiment 3, *F* = 19.90_[1,7]_, *P* = 0.003). TS MEP amplitudes were similar across TMS pulse types and tDCS_Cb_ states in experiments 2–4 (Table 1, Table 3).

### Experiment 1

Previous work had shown that short duration AP currents tended to produce MEPs with the longest latency compared with other current directions/pulse durations. This experiment shows it is true at the level of individual motor units. AP_30_ currents tended to evoke activity in SMUs that was 2–2.5 ms later than that after PA stimulation.

**Figure 4:**
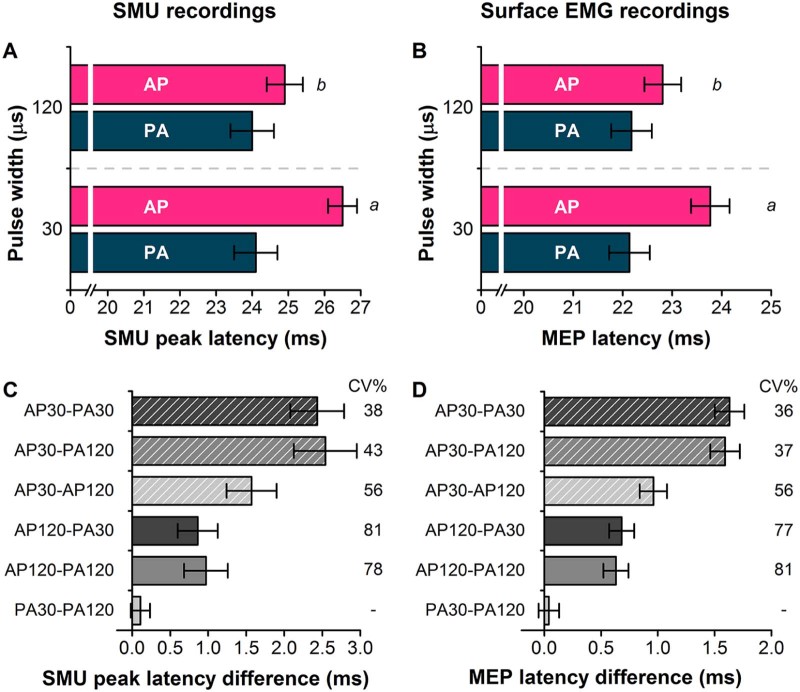
Mean latency of SMU peaks (A; Exp 1), surface EMG recorded MEP latencies (B; Exp 2), mean difference in SMU peak latencies (C; Exp 1), and surface EMG MEP latency differences (D; Exp 2). Follow up *t*-tests: *a*, *P *<* *0.001 AP_30_ versus all other pulses; *b*, *P *<* *0.001 AP_120_ versus PA_30_ and PA_120_. Inter-individual coefficient of variation (calculated as mean/SD ×100; CV%) shown for SMU peak and MEP latencies (C and D), except for PA_30_–PA_120_ where latency differences close to zero result in extremely large CV%.

Fig. 3 shows example PSTH data from 3 different units/individuals. AP_30_ currents evoked activity later than all other pulse types. Analysis of peak latency data from all 7 units showed that there were main effects of current direction (*F* = 35.05_[1,6]_, *P* = 0.001), pulse duration (*F* = 32.90_[1,6]_, *P* = 0.001) and an interaction of current direction × pulse duration (*F* = 51.66_[1,6]_, *P *<* *0.001; Fig. 4A). This is further illustrated by the differences in mean SMU peak latencies between TMS pulse types (Fig. 4C).

### Experiment 2

#### TS MEP latency

For comparison with the SMU data above, we measured TS MEP onset latencies for all pulse types during minimal voluntary contraction (Fig. 4B and D). AP_30_ currents evoked the longest latency MEPs. This was confirmed in the rmANOVA in which there were main effects of current direction (*F* = 140.4_[1,20]_, *P *<* *0.001), pulse duration (*F* = 38.2_[1,20]_, *P *<* *0.001) and an interaction of current direction × pulse duration (*F* = 44.6_[1,20]_, *P *<* *0.001). The difference in MEP onset latency of the various pulse types is similar in range to that seen in the SMUs.

#### SAI

**Figure 5:**
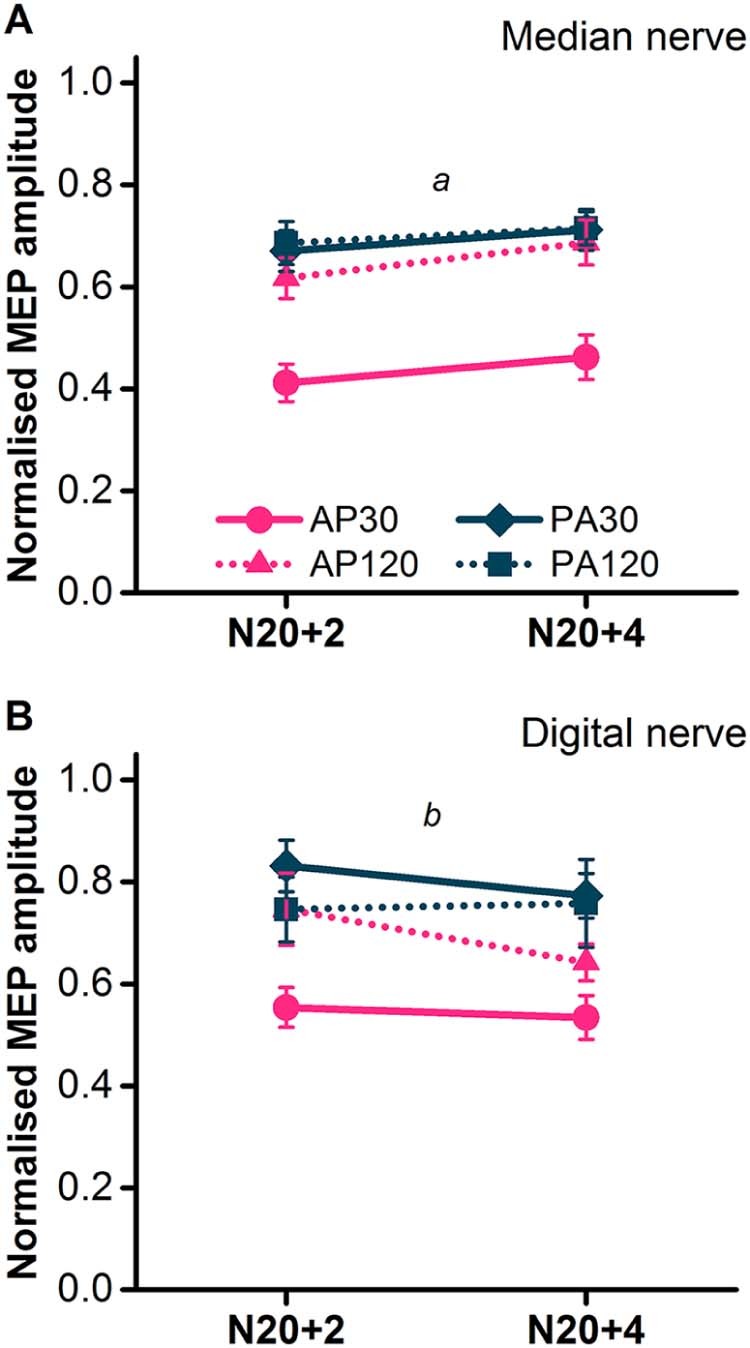
SAI assessed in the FDI during slight voluntary contraction (~10% maximum EMG) with median nerve (A; *N* = 21) and digital nerve conditioning stimuli (B; *N* = 11). N20+2 and N20+4 refer to the interval between the conditioning stimulus and test stimulus. Follow up *t*-tests: *a*, *P *<* *0.001 for AP_30_ versus all other pulses (mean of N20+2 and N20+4), *b*, *P *<* *0.01 for AP_30_ versus AP_120_ and PA_30_ (mean of N20+2 and N20+4).

AP_30_ currents were generally associated with greater median nerve SAI than all other pulse types at both ISIs (Fig. 5A). There were no differences between the three other pulses. This was supported by a significant current direction × pulse duration interaction in the rmANOVA (Table 3). Subsequent paired *t*-tests on the mean data for both ISIs revealed a significant difference between AP_30_ SAI and all other pulse types (*P *<* *0.001 all comparisons). SAI was slightly less effective with digital nerve stimulation. Nevertheless, SAI was greater for AP_30_ currents versus PA_30_ (*t*-test, *P *<* *0.001) and AP_120_ (*t*-test, *P* = 0.011), but not PA_120_ (*t*-test, *P* = 0.12) (see Fig. 5B and Table 3 for rmANOVA).

### Experiment 3

**Figure 6:**
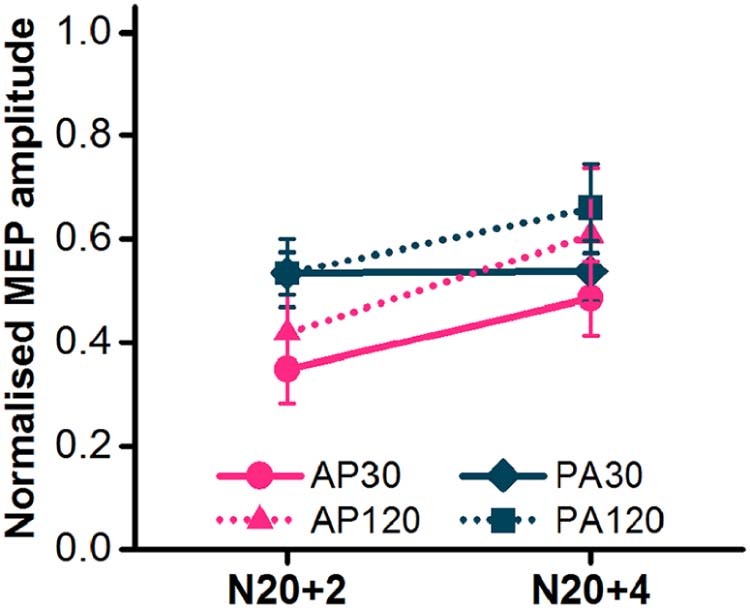
SAI assessed in the FDI at rest with median nerve conditioning stimulus (*N* = 8). N20+2 and N20+4 refer to the interval between the conditioning stimulus and test stimulus.

When SAI was tested with higher intensities in a relaxed muscle, differences in SAI between TMS pulse types were less pronounced. An interaction of current direction × ISI (Table 3, Fig. 6) appeared to reflect a greater SAI at N20+2, but not at N20+4, with AP currents compared to PA currents, but *post hoc* comparisons of the average conditioned MEP amplitude of AP_30_ and AP_120_ versus the average of PA_30_ and PA_120_ at each ISI revealed no difference between current directions at either interval (N20+2, *t*-test, *P* = 0.072; N20+4, *t*-test, *P* = 0.48).

### Experiment 4

**Figure 7:**
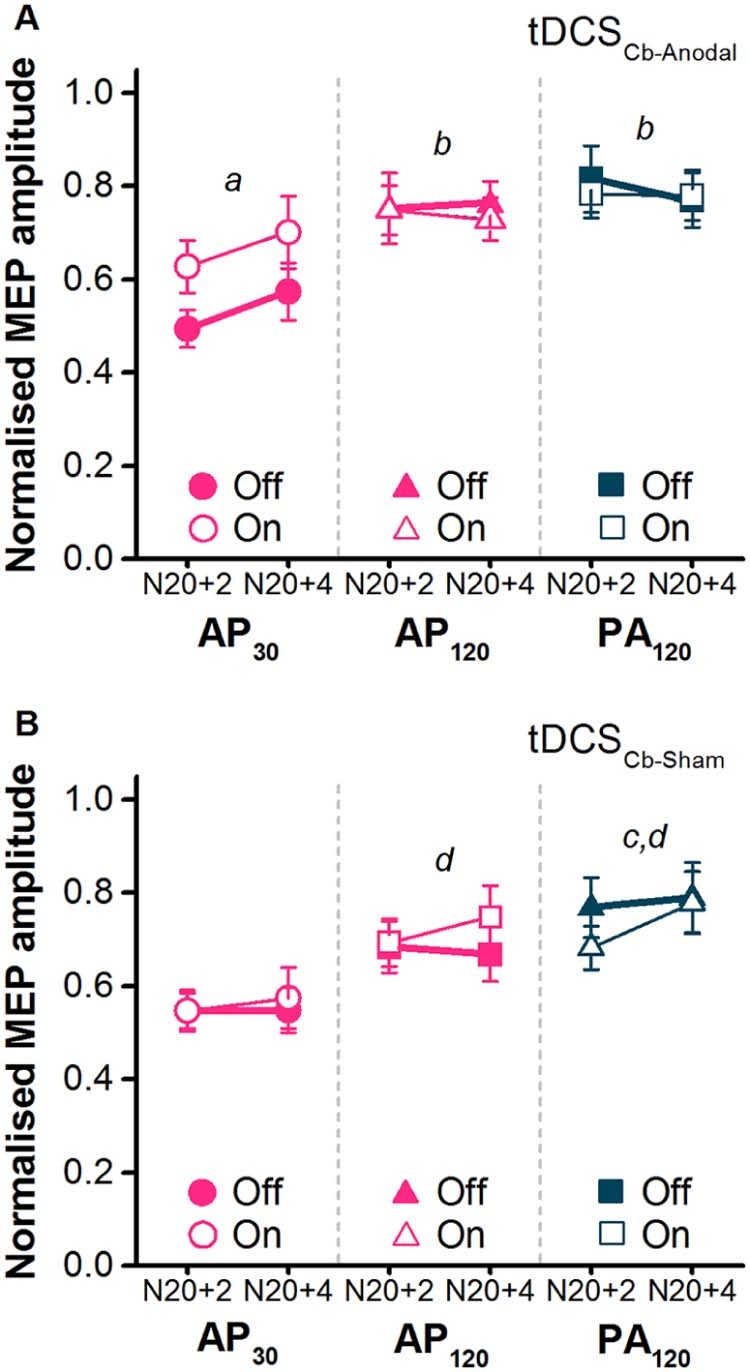
Effects of tDCS_Cb-Anodal_ (A) and tDCS_Cb-Sham_ (B) on SAI tested with median nerve conditioning stimuli and TMS test pulses comprising of different combinations of pulse duration and current direction (AP_30_, AP_120_, PA_120_). N20+2 and N20+4 refer to the interval between the conditioning stimulus and test stimulus. Off refers to baseline measurements prior to tDCS_Cb_ and On refers to measurements during. tDCS_Cb-Anodal_ follow up *t*-tests: *a*, *P *<* *0.05 for AP_30_ Off versus AP_30_ On; *b*, *P *<* *0.05 for Off state AP_30_ versus AP_120_ and PA_120_. tDCS_Cb-Sham_ follow up *t*-tests: *c*, *P *<* *0.05 for Off state AP_30_ versus PA_120_ (*P* = 0.065 for AP_30_ versus AP_120_); *d*, *P *<* *0.05 for On state AP_30_ versus PA_120_ and AP_120_.

SAI (median nerve) was reduced by tDCS_Cb-Anodal_ for the AP_30_ currents only (Fig. 7A). Indeed in the presence of tDCS_Cb-Anodal_, AP_30_ SAI was no longer different from SAI with other TMS pulse types.

In the statistics, rmANOVA on the absolute TS MEP amplitudes for both tDCS_Cb-Anodal_ and tDCS_Cb-Sham_ revealed no main effects or interactions (Table 1, Table 3), confirming that TS MEPs were not affected by tDCS_Cb_. Subsequent rmANOVA on the normalised conditioned MEP amplitudes in the tDCS_Cb-Anodal_ condition revealed a significant TMS pulse type × tDCS_Cb_ state interaction (Table 3). Subsequent *post hoc* paired comparisons on the mean data across both ISIs showed that, as in experiment 2, SAI was greater for AP_30_ in the tDCS_Cb_ Off state compared to AP_120_ and PA_120_ (*t*-tests, *P* ≤ 0.05), with no difference between the latter two pulses (*t*-test, *P* = 1.0). The new finding was AP_30_ SAI was less effective in the tDCS_Cb_ On compared with Off state (*t*-test, *P* = 0.049). Finally, there were no differences in SAI among the three TMS pulses in the tDCS_Cb_ On state (*t*-tests all *P* ≥ 0.35).

In the tDCS_Cb-Sham_ condition, rmANOVA showed no main or interaction effect of tDCS_Cb_ state, indicating that it had no effect on SAI for any TMS pulse type (Table 3; Fig. 7B).

To compare the effects of real and sham tDCS_Cb_, we calculated the percentage change in SAI from Off to On in the two conditions. rmANOVA showed no main effects of TMS pulse type (*F*_[2,20]_ = 2.85, *P* = 0.081) or tDCS_Cb_ condition (*F*_[1,10]_ = 2.96, *P* = 0.116), but did show a significant interaction of TMS pulse type × tDCS_Cb_ condition interaction (*F*_[2,20]_ = 7.52, *P* = 0.004). The change in SAI was greater for AP_30_ currents in the tDCS_Cb-Anodal_ condition versus tDCS_Cb-Sham_ (31 ± 8% versus −4 ± 5%; *t*-test *P* = 0.02), further illustrating the effect of tDCS_Cb-Anodal_. There were no differences between tDCS_Cb_ conditions for AP_120_ or PA_120_ currents (*t*-tests, *P* ≥ 0.72).

## Discussion

### Selective recruitment of late inputs by AP_30_ currents

It is well-known that different current directions recruit SMUs and MEPs with different latencies. This is because AP currents activate later arriving excitatory I-wave inputs which in turn initiate descending activity in corticospinal neurons some 2–3 ms later than that evoked by PA currents [2], [3]. In line with our recent findings [10], we show here that pulse duration also influences the latency of responses, since MEPs elicited by AP_30_ currents during background voluntary contraction had a longer average latency than all other pulse types, including AP_120_ currents (Fig. 4A and C). One previous study found no effect of pulse duration on MEP latencies [11], but this could potentially be explained by the narrow range of pulse durations (82–114 µs) employed and assessment of only PA evoked responses at rest.

The present data exclude the possibility that the difference in latencies occurs because AP_30_ currents recruit slower conducting spinal motoneurons since the same latency difference is present within individual SMUs. Thus, AP_30_ currents evoked peaks of increased SMU firing which were usually several milliseconds longer than those evoked by PA_120_ currents whilst AP_120_ currents seemed to evoke a mixture of the two. In a previous paper we had speculated [10] that an AP_30_ pulse tends to favour recruitment of I_3_ waves whereas AP_120_ currents are less selective and may also activate the early I_1_-waves recruited by PA_120_ and PA_30_ currents. We proposed that use of AP_30_ currents may help achieve a better distinction than use of AP_120_ currents between the recruitment of early PA-sensitive inputs and the late AP-sensitive inputs. However, the new results in the present paper suggest that AP_30_ and AP_120_ currents activate *different* late inputs to corticospinal neurons.

Interestingly, the 2–3 ms SMU latency difference between AP_30_ and PA currents we report here is similar to the AP–PA difference reported with standard (82 µs) pulses [2], seeming to suggest that AP_30_ currents recruit the same I_3_ inputs as standard AP pulses. Again, however, the results discussed below appear to argue against this, and imply that AP_30_ currents might activate a different population of late inputs to standard AP currents.

### AP_30_-sensitive late inputs are more responsive to SAI

AP_30_ MEPs evoked in actively contracting muscle were suppressed more by peripheral nerve inputs (SAI) than those evoked by AP_120_, PA_30_ and PA_120_ currents, and this was generally consistent for both cutaneous and mixed afferent sources of somatosensory input (Fig. 5A and B). The original report of corticospinal activity and SMU responses suggested that late CSN inputs (I_3_-waves) are more susceptible to afferent inhibition than early inputs when using standard PA currents at rest [15]. If one were simply to assume that same late (I_3_-wave) inputs are recruited by AP_30_, AP_120_, PA_30_ and PA_120_ currents, the interpretation would be that AP_30_ currents recruit proportionately more late inputs than the other pulse types and hence reveal the extent of SAI which may otherwise be obscured by concomitant recruitment of earlier inputs that are less susceptible to inhibition, as is likely with other pulse types (PA_120_, PA_30_, AP_120_).

An alternative explanation follows the suggestion put forward in several studies that different interneuron circuits are responsible for late activity evoked by each current direction [3], [4], [8], [22]. Ni et al. [8] found that, using TMS pulses, late peaks in SMU recordings (I_3_ waves) recruited by AP currents were less susceptible to SAI than late PA-recruited peaks. The implication was that not all late I-wave generators are the same. At first glance the data of Ni et al. seem inconsistent with ours, since we found greater SAI for AP_30_ currents compared to PA. However, their study was conducted at rest and they reported no difference in the effect of SAI on AP- and PA-evoked MEPs when they tested during contraction (see their Fig. 7). We found the same for more typical long duration pulses, whereby PA_120_- and AP_120_-evoked MEPs responded similarly to SAI during contraction. The difference here is that AP_30_-evoked MEPs were more sensitive to SAI, possibly because these pulses recruit a different set of late inputs to those recruited by long duration PA and AP currents, and potentially different from those recruited by standard AP currents as used by Ni et al. [8].

Any advantage of using AP_30_ currents may be lost if SAI is evaluated at rest. Data collected in the relaxed FDI showed minimal differences between TMS pulses in the level of SAI (Fig. 6). This is probably because resting MEPs have a higher threshold and therefore require a higher stimulus intensity which may activate a mixture of activity from a variety of early and late CSN inputs [2], [3], [4]. Another possibility is that the inputs recruited by different pulses vary in their interaction with combined afferent input and voluntary muscle activity. The effect of voluntary contraction on the level of SAI tested with AP_30_ currents was seemingly minimal (see Figure 5, Figure 6), whereas it seemed to attenuate SAI tested with PA and AP_120_ currents (Figure 5, Figure 6). Thus the inputs recruited by PA and AP_120_ currents may be more sensitive to voluntary contraction than those recruited by AP_30_ currents, again suggesting that AP_30_ currents recruit a different set of inputs.

### tDCS_Cb-Anodal_ interacts with SAI tested with AP_30_ currents

Anodal tDCS_Cb_ has been reported to have no after-effect on SAI when tested with PA currents at rest in healthy volunteers [17], [23]. However, abnormal SAI has been found in patients with cerebellar degeneration [12], and also in patients with Alzheimer's disease where it has been shown to be partially dependent on cerebellar function [13]. Furthermore, more detailed investigation in a healthy population revealed that modulation of cerebellar activity via tDCS_Cb-Anodal_ specifically reduces the excitability of late AP-sensitive inputs during stimulation [7]. These data imply there is a functional connection between the cerebellum and late AP-sensitive, but not PA-sensitive, inputs in M1. Based on these studies we asked whether SAI tested with low intensity AP_120_, AP_30_ and PA_120_ currents might be differentially altered during tDCS_Cb-Anodal_. The data indicated a small reduction in SAI for AP_30_ currents during tDCS_Cb-Anodal_, but not during tDCS_Cb-Sham_ (Fig. 7). The present data therefore suggest that the cerebellar activity specifically modulates the inhibitory effect of SAI on AP_30_-sensitive inputs to CSNs.

tDCS_Cb-Anodal_ could have affected SAI in a number of ways. Previous studies found no change in the early components somatosensory evoked potentials (N20, P25) or early high-frequency oscillations after tDCS_Cb-Anodal_ [17], suggesting that the intervention is unlikely to have affected processing of afferent signals within the somatosensory cortex or thalamus. However it is possible that the same sensory signal could interact differentially with distinct CSN inputs in M1, and that one of these interactions is regulated by input from the cerebellum arriving via the cerebello–thalamo–cortical pathway. For example, early and late CSN inputs might arrive at different locations on the dendritic tree and these locations could be differentially sensitive to inhibition from the interneurons responsible for SAI. Such branch-specific inhibition of pyramidal neurons has been reported in animal experiments [24]. Tonic activity in a cerebello–thalamo–cortical pathway could facilitate cortical inhibitory interneurons mediating SAI onto late inputs targeted by AP_30_ currents, and this facilitation may be removed by cerebellar direct current stimulation.

We note that previous studies had investigated the effects of tDCS_Cb-Anodal_ on the after-effects of a paired-associative stimulation (PAS) protocol [7], [17], which involves the pairing of a peripheral electrical stimulus and TMS pulse over M1 20–25 ms later, and is used to induce a long-lasting (up to 30 min) facilitation of M1 excitability [25]. However, we think that this effect is unlikely to be related to the effect we observed on SAI. Afferent input appears to have two actions on M1: one is to inhibit MEPs [15] and the other is a parallel excitatory effect (e.g. short-interval intracortical facilitation is enhanced in its presence [21]). If one assumes that the excitatory effect is mainly responsible for the PAS after-effect [21], whereas the inhibitory effect is responsible for SAI, then the data would be compatible with the idea that they could differ in their interaction with input from cerebellum in terms of both the M1 inputs targeted and the timing of the interaction.

There are several potential caveats to our interpretation of these results. Cerebellar tDCS is increasingly being used to study the physiology and function of the cerebellum [6]. Several lines of evidence suggest tDCS_Cb_ is likely to influence the excitability of some neural elements in the cerebellum with relatively little activation of neighbouring areas of the brain. Firstly, modelling of the electric field suggests it is maximal in the cerebellar hemisphere under the electrode, with little spread to other nearby structures [26]. Secondly, tDCS_Cb_ has been reported to modulate cerebellar-evoked inhibition of M1 in a polarity-dependent manner for a brief period following stimulation, without appearing to affect measures of M1, brainstem or spinal excitability [16]. Thirdly, although stimulation of cutaneous sensory receptors under the tDCS electrodes remains a possibility, it seems unlikely that it could explain the present effects on SAI in the hand muscles. Animal studies show that the somatosensory input reaching M1 output cells via S1 appears to display strong somatotopy [27], [28] and the sensorimotor interactions tested by SAI in humans also exhibit a strong somatotopy [5], [29]. There remains, however, some uncertainty surrounding exactly which structures are targeted by tDCS_Cb_ and the mechanisms by which it might interact with the cerebellum [6]. Furthermore, we acknowledge that online changes in cerebellar excitability could have been influenced by the concurrent muscle contraction and long duration of the tDCS_Cb_ given that both factors are known to influence the after-effects of tDCS on M1 excitability [30], [31].

## Conclusions

The potential to manipulate pulse duration represents a new dimension of control in TMS. The use of brief AP currents enabled the selective recruitment of the longest latency MEPs and this conferred a benefit when assessing SAI by revealing greater inhibition compared to PA and long duration AP currents. Further, it helped reveal an influence of the cerebellum on SAI onto late AP_30_-sensitive corticospinal inputs. The inputs targeted by brief AP currents appear distinct from those targeted even by long duration AP and PA pulses, and thus pulse duration appears an important determinant of what is activated with TMS in human motor cortex.

## Figures and Tables

**Table 1:** Test stimulus (TS) intensities and MEP amplitudes for each experiment (mean ± SEM).

	TMS pulse combination (current direction and pulse duration)			
	AP_30_	PA_30_	AP_120_	PA_120_
Experiment 1 (*N* = 7)				
TS intensity (%)	78 ± 6	61 ± 5	31 ± 2	26 ± 2
Experiment 2 (median nerve stimulation, *N* = 21)				
TS intensity (%)	77 ± 3	58 ± 3	34 ± 1	26 ± 1
TS MEP amplitude (mV)	0.99 ± 0.06	1.09 ± 0.08	1.08 ± 0.05	1.04 ± 0.05
Experiment 2 (digital nerve stimulation, *N* = 10)				
TS intensity (%)	77 ± 4	59 ± 4	31 ± 2	26 ± 2
TS MEP amplitude (mV)	1.10 ± 0.08	1.06 ± 0.06	1.01 ± 0.07	1.02 ± 0.03
Experiment 3 (*N* = 8)				
TS intensity (%)	81 ± 4	67 ± 3	34 ± 1	29 ± 1
TS MEP amplitude (mV)	1.32 ± 0.13	1.22 ± 0.13	1.30 ± 0.11	1.31 ± 0.09
Experiment 4 (*N* = 11)				
Anodal tDCS_Cb_				
TS intensity (%) Off	81 ± 3	–	34 ± 1	27 ± 1
TS MEP amplitude (mV) Off	1.21 ± 0.07	–	1.13 ± 0.06	1.08 ± 0.03
TS intensity (%) On	81 ± 3	–	34 ± 1	27 ± 1
TS MEP amplitude (mV) On	1.28 ± 0.07	–	1.20 ± 0.09	1.09 ± 0.05
Sham tDCS_Cb_				
TS intensity (%) Off	83 ± 1	–	35 ± 1	28 ± 1
Test MEP amplitude (mV) Off	1.17 ± 0.05	–	1.15 ± 0.06	1.10 ± 0.05
TS intensity (%) On	83 ± 1	–	34 ± 1	28 ± 1
TS MEP amplitude (mV) On	1.24 ± 0.07	–	1.22 ± 0.07	1.14 ± 0.06

**Table 2:** Sensory thresholds (ST), conditioning stimulus (CS) intensities and N20 latencies in experiments 2–4 (mean ± SEM).

	Median nerve CS				Digital nerve CS			
	ST (mA)	CS (mA)	CS/ST	N20 latency (ms)	ST (mA)	CS (mA)	CS/ST	N20 latency (ms)
Experiment 2	2.3 ± 0.2	5.5 ± 0.5	2.4 ± 0.1	19.7 ± 0.3	3.0 ± 0.4	9.1 ± 1.1	3.0 ± 0.0	22.7 ± 0.6
Experiment 3	1.9 ± 0.2	4.5 ± 0.6	2.4 ± 0.2	20.0 ± 0.4	–	–	–	–
Experiment 4								
tDCS_Cb-Anodal_	1.9 ± 0.2	4.0 ± 0.2	2.3 ± 0.2	19.2 ± 0.4	–	–	–	–
tDCS_Cb-Sham_	1.9 ± 0.1	4.2 ± 0.3	2.2 ± 0.1	19.2 ± 0.4	–	–	–	–

**Table 3:** Results for ANOVAs performed to evaluate differences in absolute TS MEP amplitude and normalised CS MEP amplitudes across TMS pulse types for each experiment. *F*_[DF,error]_ and *P* are reported. Significant main effects and interactions are indicated by bold values.

Experiment 2	Absolute TS MEP amplitude				Normalised conditioned MEP amplitude			
	Median nerve CS		Digital nerve CS		Median nerve CS		Digital nerve CS	
	*F*_[DF,error]_	*P*	*F*_[DF,error]_	*P*	*F*_[DF,error]_	*P*	*F*_[DF,error]_	*P*
Pulse duration	0.04_[1,20]_	0.680	1.09_[1,10]_	0.321	**40.59**_[1,20]_	**<0.001**	1.07_[1,10]_	0.325
Current direction	0.18_[1,20]_	0.841	0.03_[1,10]_	0.867	**26.12**_[1,20]_	**<0.001**	**11.90**_[1,10]_	**0.006**
ISI		–		–	**4.97**_[1,20]_	**0.037**	1.63_[1,10]_	0.231
Pulse duration × current direction	1.53_[1,20]_	0.231	0.43_[1,10]_	0.525	**30.26**_[1,20]_	**<0.001**	**13.71**_[1,10]_	**0.004**
Pulse duration × ISI		–		–	2.40_[1,20]_	0.137	0.03_[1,10]_	0.863
Current direction × ISI		–		–	0.01_[1,20]_	0.911	0.78_[1,10]_	0.399
Pulse duration × current direction × ISI		–		–	0.23_[1,20]_	0.226	1.71_[1,10]_	0.222

Experiment 3	Median nerve CS				Median nerve CS			
	*F*_[DF,error]_	*P*	*F*_[DF,error]_	*P*	*F*_[DF,error]_	*P*	*F*_[DF,error]_	*P*
Pulse duration	0.16_[1,7]_	0.703		–	2.65_[1,7]_	0.184		–
Current direction	0.13_[1,7]_	0.727		–	2.17_[1,7]_	0.147		–
ISI		–		–	1.93_[1,7]_	0.207		–
Pulse duration × current direction	0.25_[1,7]_	0.630		–	0.11_[1,7]_	0.747		–
Pulse duration × ISI		–		–	1.29_[1,7]_	0.293		–
Current direction × ISI		–		–	**7.27_[1,7]_**	**0.031**		–
Pulse duration × current direction ×ISI		–		–	0.34_[1,7]_	.578		–

Experiment 4	Median nerve CS		Median nerve CS		Median nerve CS		Median nerve CS	
	tDCS_Cb-Anodal_		tDCS_Cb-Sham_		tDCS_Cb-Anodal_		tDCS_Cb-Sham_	
	*F*_[DF,error]_	*P*	*F*_[DF,error]_	*P*	*F*_[DF,error]_	*P*	*F*_[DF,error]_	*P*
TMS pulse type	2.38_[1.3,13.4]_	0.142	1.61_[2,20]_	0.226	**7.58**_[1.3,12.7]_	**0.013**	**9.04**_[2,20]_	**0.002**
tDCS_Cb_ state	3.82_[1,10]_	0.079	3.10_[1,10]_	0.109	**6.0**_[1,10]_	**0.034**	0.03_[1,10]_	0.869
ISI		–		–	2.25_[1,10]_	0.165	1.87_[1,10]_	0.202
TMS pulse type × tDCS_Cb_ state	0.30_[2,20]_	0.747	0.38_[2,20]_	0.689	**4.53**_[2,20]_	**0.024**	1.56_[2,20]_	0.235
TMS pulse type × ISI		–		–	0.99_[2,20]_	0.388	0.18_[2,20]_	0.834
tDCS_Cb_ state × ISI		–		–	0.02_[1,10]_	0.891	**11.66_[1,10]_**	**0.007**
TMS pulse type × tDCS_Cb_ state × ISI		–		–	0.66_[2,20]_	0.529	2.03_[2,20]_	0.157
